# Do Obsessive-Compulsive Symptoms Increase the Risk of Developing Psychosis? A Systematic Review and Meta-analysis

**DOI:** 10.1093/schizbullopen/sgag011

**Published:** 2026-05-12

**Authors:** William Hinton, Marco Vivolo, Emma Jimenez, Joanne Hodgekins

**Affiliations:** Department of Clinical Psychology and Psychological Therapies, Norwich Medical School, Faculty of Medicine and Health Sciences, University of East Anglia, Norwich Research Park, Norwich, Norfolk NR4 7TJ, United Kingdom; Cambridge and Peterborough Foundation Trust Elizabeth House, Fulbourn, Cambridge CB21 5EF, United Kingdom; Department of Clinical Psychology and Psychological Therapies, Norwich Medical School, Faculty of Medicine and Health Sciences, University of East Anglia, Norwich Research Park, Norwich, Norfolk NR4 7TJ, United Kingdom; Cambridge and Peterborough Foundation Trust Elizabeth House, Fulbourn, Cambridge CB21 5EF, United Kingdom; Department of Clinical Psychology and Psychological Therapies, Norwich Medical School, Faculty of Medicine and Health Sciences, University of East Anglia, Norwich Research Park, Norwich, Norfolk NR4 7TJ, United Kingdom; Department of Clinical Psychology and Psychological Therapies, Norwich Medical School, Faculty of Medicine and Health Sciences, University of East Anglia, Norwich Research Park, Norwich, Norfolk NR4 7TJ, United Kingdom

**Keywords:** OCD, schizophrenia, longitudinal, clinical high risk, transition, conversion

## Abstract

**Background and Hypothesis:**

There is a phenomenological overlap of obsessive-compulsive symptoms (OCSs) and psychosis, leading to discussion about whether OCSs increase the risk of developing psychosis. Previous reviews have focused on clinical high-risk (CHR) cohorts, which may misestimate risk due to sampling biases. The current systematic review and meta-analyses aimed to determine the risk of OCSs on developing psychosis in individuals classified at CHR and at the population level.

**Study Design:**

A total of 2081 articles were screened, with 11 studies meeting the criteria for inclusion. Two separate meta-analyses were conducted for CHR cohorts and for register-based cohorts to estimate the risk ratio (RR) of OCSs for developing psychosis.

**Study Results:**

In CHR cohorts, the meta-analysis found no significant difference in the risk of developing psychosis between individuals with and without OCSs (RR = 0.99, 95% CI, 0.71-1.38, *P* = .95) across 8 studies with low heterogeneity (*I*^2^ < 0.000%, 95% CI, 0-67.03). In register-based cohorts, OCSs were associated with a 15-fold increase in the risk of developing psychosis (RR = 15.01, 95% CI, 8.36-26.93, *P* < .001), although this estimate was derived from 3 studies with significant heterogeneity (*I*^2^ = 85.1%, 95% CI, 37.14-99.67).

**Conclusions:**

The limited number of studies and high heterogeneity in the population-level cohort limit any firm conclusions. However, the findings indicate that OCSs may increase the risk of developing psychosis in the register-based cohorts, but not in CHR cohorts. The discrepancy may reflect shared underlying vulnerabilities present in both OCSs and psychosis that are obscured by samples enriched for psychosis risk.

## Introduction

A wealth of research has been dedicated to identifying and preventing psychosis in the early stages to reduce the severity of illness and burden of the disease.^1^^,^^2^ The duration of untreated psychosis is associated with poorer functional outcome, treatment resistance, higher rates of relapse, and iatrogenic harm,^3^ giving rise to the critical-period hypothesis that the early phase of psychosis offers a crucial opportunity for intervention.^4^ Early intervention in psychosis (EIP) services that provide support during this critical period have demonstrated effectiveness in improving symptom severity, functioning, and treatment adherence, highlighting the importance of early detection.^5^

This led to the development of the clinical high-risk (CHR) paradigm to identify individuals at increased risk of psychosis and offer preventative intervention.^6^ Classification typically includes attenuated psychotic symptoms (APSs), basic symptoms (eg, perceptual or cognitive disturbances), or genetic risk and functional decline.^7^ Tools such as the Structured Interview for Psychosis-Risk Syndromes (SIPS) and Comprehensive Assessment of At-Risk Mental States (CAARMS) have been validated for identifying people at CHR showing high sensitivity but low specificity, meaning that those who develop psychosis are likely to test positive for psychosis risk, but two-thirds of those who screen positive do not transition.^8–10^

Many of those at CHR who do not develop psychosis will experience persistent symptoms, poorer functioning, and comorbid anxiety or depression, suggesting that CHR may reflect vulnerability to broader psychopathology.^11^^,^^12^ Conversely, psychosis may have a heterotypic course where, for example, nonpsychotic symptoms evolve into psychotic symptoms.^13^ Although comorbid mood and anxiety disorders in CHR youth do not appear to predict transition,^14^ some clinical risk syndromes for emerging nonpsychotic conditions (eg, bipolar disorder) are associated with an elevated risk of psychosis compared to the general population.^15^ The Clinical High At-Risk Mental States (CHARMS) framework recognizes multiple pathways to and from the CHR state, describing overlapping exit syndromes such as bipolar, depressive, and personality disorders, where subthreshold symptoms precede full disorder.^16^

There is also considerable overlap between obsessive-compulsive symptoms (OCSs) and psychosis.^17^ Like psychosis, people with obsessive-compulsive disorder (OCD) report subthreshold symptoms several years prior to meeting diagnostic thresholds,^18^^,^^19^ and OCSs share multiple overlaps with psychosis, including genetic risk,^20–22^ brain abnormalities,^23^ early adversity,^24^^,^^25^ and cognitive and psychological mechanisms such as magical thinking,^26–28^ intolerance of uncertainty,^29^^,^^30^ inflated sense of responsibility,^31^^,^^32^ and source monitoring errors.^33^^,^^34^

In addition to the overlap in clinical characteristics, OCS and psychosis also conceptually overlap.^35^ Early phenomenological research defined obsessions as strictly requiring resistance, although the criteria have loosened since the 1980s.^36^ This reflects contemporary debate on the requirement of insight for a diagnosis of OCD, with disagreement whether the “absent or delusional insight” specifier should be classed as a specifier or an additional diagnosis.^37^ This overlap presents a theoretical and clinical challenge that obscures clear boundaries between OCSs and psychosis,^17^ and what constitutes transition.^38^

Due to the overlap, it has been proposed that OCSs may form part of a risk profile for psychosis.^39^ In pediatric OCD, earlier age of onset, greater OCD severity, and poorer insight have all been associated with psychotic vulnerability.^40^^,^^41^ However, findings from studies examining transition rates are mixed, with studies of CHR cohorts finding no association,^42^ an increased risk,^43^ and a protective effect.^44^ Based on a narrative synthesis of four studies, a systematic review found that OCSs did not increase the risk of developing psychosis.^45^ Similarly, another review and meta-analysis analyzed risk across 5 studies (3 of which were included from the previous review) and found that OCSs did not increase the risk of developing psychosis, with an odds ratio of 0.82 (95% CI 0.42-1.61).^46^ In contrast, register-based studies using nationwide population data have consistently found an elevated risk of psychosis.^22^^,^^47^

The contrasting findings may reflect methodological issues such as sampling biases leading to an overrepresentation of help-seeking people with multiple comorbidities but low psychosis risk and underrepresentation of people who developed psychosis but were never identified as CHR.^14^^,^^38^ For example, there is evidence that only 4%-5% of the people presenting to secondary care with psychosis had previously been in contact with the local CHR service,^48^^,^^49^ and only ~40% of individuals with OCD seek treatment,^50^ and those who do are often seen by other teams (eg., community mental health or inpatient teams). Consequently, people with OCSs may be underrepresented in CHR samples, obscuring their true risk contribution.

The present systematic review and meta-analyses aimed to determine the risk posed by OCSs in developing psychosis among CHR participants, and provide an update on previous reviews as well as including population-level data that capture the experiences of people with OCSs who proceed to develop psychosis that may not be captured in CHR studies.

## Methods

A systematic review protocol was developed in line with Preferred Reporting Items for Systematic Reviews and Meta-Analyses (PRISMA) guidelines.^51^ The protocol was registered on the International Register of Prospective Systematic Reviews (PROSPERO; CRD420250653682, 05/03/2025) to ensure the transparency of the research, rationale, objectives, methods, and process of data analysis.

### Eligibility

#### Inclusion Criteria

For inclusion, studies must have (1) measured baseline OCSs by a validated questionnaire or standardized diagnostic criteria (eg, structured interview or as part of routine clinical assessment); (2) a control group with an absence of baseline OCSs measured by a validated questionnaire or standardized diagnostic criteria; (3) data on the number of people in the exposure and control groups who did and did not develop psychosis measured by a validated questionnaire or structured diagnostic interview; (4) psychosis or schizophrenia measured by a validated questionnaire or standardized diagnostic criteria; and (5) longitudinal data of at least 6 months between measurements. The current review focused on articles from 2005 to capture recent estimates of the risk of developing psychosis using contemporary assessment methods, such as the CAARMS and SIPS, which were validated and more widely used after this period.^52^^,^^53^ The increased standardization of measures reduces heterogeneity and enhances methodological comparability.

#### Exclusion Criteria

Studies were excluded if (1) the follow-up period was shorter than 6 months; (2) the article was a case report or not peer reviewed; (3) part or all of the data were missing; or (4) there was no full text or English language article available. The follow-up period of 6 months was chosen as shorter intervals are less likely to observe transition events, which can lead to underpowered studies.^54^ However, the selection of a follow-up period was balanced with the aim to capture faster transitions as just under 10% will occur within 6 months.^10^

### Search Strategy

Standardized search terms were used on PsycINFO, PubMed, and OVID indexed from 2005 to 2025. The search terms were the following: ((“OCD” OR “obsessive-compulsive disorder” OR “OCS”) AND (“psychosis” OR “psychotic” OR “schizophrenia” OR “schizo*”) AND (“conversion” OR “transition” OR “prodrome” OR “predict*” OR “risk”)). The search was conducted on May 20, 2025. Backward and forward reference mining was also conducted to identify additional studies not found from the database search.

### Data Extraction

The lead author (W.H.) extracted data from the included studies using the PRISMA guidelines. The following data were extracted: author, year of publication, location, study design, participant type, sample size, data collection period, follow-up period, age and gender of the sample (by group or, if this was not available, from the whole sample), measures of OCS and psychosis, and proportion of control and exposed participants who had OCD at baseline and psychosis at follow-up.

### Data Analysis

Data were analyzed using relative risk ratios (RRs) of the proportion of experimental and control groups that developed and did not develop psychosis. The RR was chosen as the preferred effect size for longitudinal data because it provides a more interpretable measure of risk than the odds ratio.^55^ Risk ratios were calculated from four datapoints: the number of people with and without OCSs who did or did not develop psychosis. A pooled RR of 1 indicates no difference in risk, an RR greater than 1 indicates increased risk, and an RR below 1 indicates reduced risk or a protective effect. This is reported alongside 95% CIs and *P*-values.

Although the initial protocol specified a single meta-analysis with subgroup analyses, two meta-analyses were conducted because of the distinct populations used in studies. One set of studies compared the risk of psychosis in CHR cohorts, while the other investigated the risk in register-based cohorts. Due to the differing sampling frames, it would be inappropriate to pool an effect size. Power analysis suggests that to detect an RR of 1.3 at *α* = .05 and power = .8 with a transition rate of 25%,^10^ 1145 participants would be required.

Due to the expected heterogeneity between studies, a random-effects model was used in both analyses. Heterogeneity was assessed using Cochran’s *Q*, *I*^2^, tau, and tau-squared for a thorough overview. An *I*^2^ of 25% or below indicates low heterogeneity, 50% indicates moderate heterogeneity, and above 75% indicates high heterogeneity.^56^ As both meta-analyses had fewer than 10 studies, resulting in being underpowered, funnel plot inspection and Egger’s test were not conducted, as recommended.^57^ Outlier analysis was conducted for both meta-analyses, and leave-one-out analysis was conducted for the meta-analysis of the CHR cohort.

All the data were analyzed using RStudio (version 2025.05.1+513) using the “metafor” package.^58^ Outlier analyses were conducted using the “dmetar” package.^59^

### Quality Appraisal

The quality of the included articles was assessed using the Newcastle-Ottawa Quality Assessment Form for Cohort Studies (NOS), which can be used to assess the risk of bias in non-randomised and observational studies. The NOS is a validated and widely used tool for assessing methodological quality.^60^ The NOS categorizes studies into good, fair, or poor quality by meeting criteria for stars across selection, comparability, and outcome. A second author appraised 3 of the 11 included studies for interrater reliability. The raters discussed any discrepancies to agree a resolved score. A follow-up period of more than a year was considered adequate for the outcome section based on previous research finding that more than half of those who do develop psychosis after being classified as CHR will transition by one year.^10^

## Results

### Study Selection

**Figure 1:**
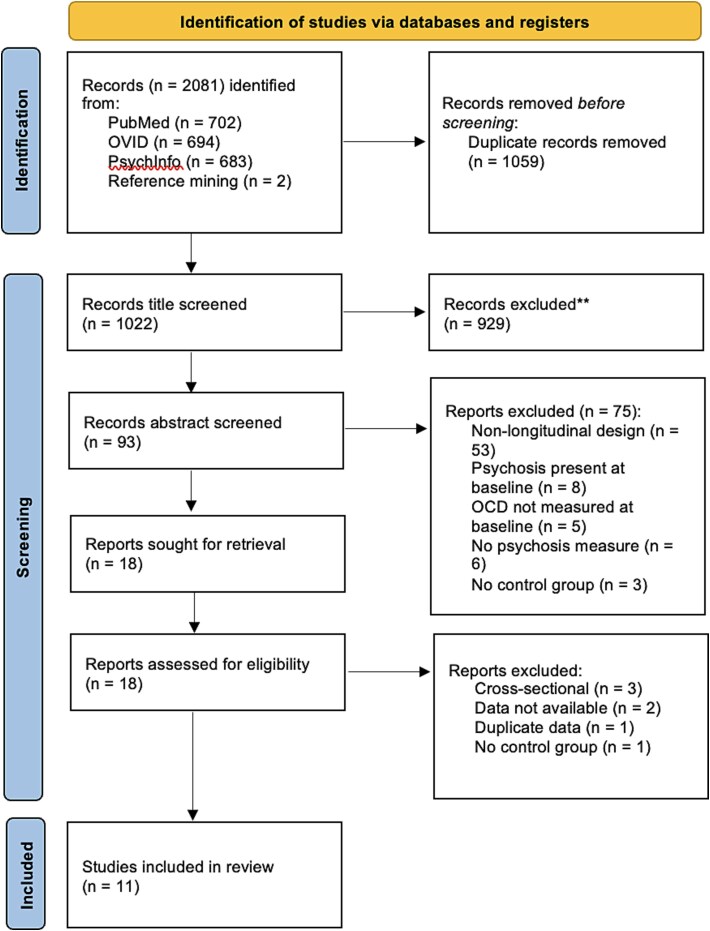
PRISMA Flow Chart of Study Selection Process

Of the 2079 papers screened, 11 were included for analysis, as shown in Figure 1. Title screening was conducted by one author (W.H.). A second reviewer (E.W.) screened 50% of the articles at the abstract stage with an interrater reliability of 93% (Cohen’s *k* = 0.76), indicating substantial agreement. Of the 25% of full-text articles screened by the same reviewers as the abstract stage, there was complete agreement.

Two authors were contacted to obtain data, but these were unavailable. One study, which used a register of over 3 million Danish people, calculated an incidence RR of 6.9 (95% CI, 6.25-7.6) for 16 231 people who developed schizophrenia, of which 447 had a prior hospital contact for OCD.^22^ Another study used a multistage, stratified, random-sampling procedure to identify 7076 Dutch participants from the general population (excluding those in institutions), and found that OCD at baseline was associated with an odds ratio of 9.4 (95% CI, 1.1-79.6), which was significant after controlling for psychosis symptoms and anxiety disorders at baseline, age, sex, education, marital status, substance use, and urbanicity.^61^

### Study Characteristics

Of the 11 articles included in the review, 8 were prospective studies and 3 were retrospective studies. The 8 prospective studies used a CHR cohort, and the 3 retrospective studies used national registers. The studies were conducted from the USA (*k* = 5), South Korea (*k* = 2), Australia, Italy, Sweden, and Taiwan (all *k* = 1).

There were 1 303 participants across the CHR studies, ranging from 20 to 352 participants in individual studies. The average follow-up period was 2.64 years (SD = 1.96), ranging from 11 months to 7.4 years. OCSs were measured by the Structured Clinical Interview for Diagnostic and Statistical Manual of Mental Disorders (SCID) in 7 studies and the Diagnostic Interview for Genetic Studies (DIGS) in 1 study. Psychosis transition was measured by SCID in 4 studies, SIPS in 3 studies, and the CAARMS in 1 study. The average transition rate in the CHR studies was 19.26%.

There were 236 781 participants in the register-based studies, ranging from 10 036 to 216 700 participants. The average follow-up period was 4.81 years (SD = 2.2), ranging from 2.4 to 6.7 years. All the psychosis and OCD measures were using International Classification of Diseases (ICD) codes, with one study using ICD-9, another using ICD-10, and the final study using ICD-10 for OCD and ICD-8 to ICD-10 for psychosis. The average transition rate in the register-based studies was 0.52%.

### Participant Characteristics

Further demographic information is summarized in Table 1 alongside proportions of people who had OCSs at baseline and how many developed psychosis.

**Table 1:** Study and Participant Characteristics^a^

**Study**	**Sample size**	**Data collection period**	**Follow-up period (years)**	**NOS rating**	**Mean age (SD)**		***n* females (%)**		***n***			
					**OCD+**	**OCD−**	**OCD+**	**OCD−**	**P + OCD+**	**P − OCD+**	**P + OCD−**	**P − OCD−**
**CHR^b^**												
Fontenelle et al. (2011);^42^ Australia	312	1994-2005	7.4	Poor	19.3 (3.1)	19.9 (3.3)	16 (61)	158 (54.9)	4	22	34	252
Niendam et al. (2009);^44^ USA	64	N/K	0.92	Poor	15.58 (2.03)	16.68 (2.39)	6 (46)	19 (37)	0	13	11	40
De Vylder et al. (2012);^62^ USA	20	N/K	3.44	Poor	21.2 (3.1)	20.1 (4.8)	5 (41.7)	3 (37.5)	3	9	2	6
Hur et al. (2012);^63^ South Korea	53	2004-2010	1	Poor	20.79 (3.96)	20.95 (3.81)	10 (41.7)	15 (36.6)	3	15	7	28
Alessandro et al. (2024);^64^ Italy	180	2016-2021	2	Poor	19.55 (3.78)	19.52 (3.82)	28 (42.4)	62 (54.5)	11	55	18	96
Kennedy et al. (2021);^65^ USA	122	N/K	2	Poor	19.83 (3.78)		58 (37)		5	16	34	67
Brucato et al. (2017);^43^ USA	200	2003-2015	2	Poor	20.03 (3.85)		54 (27.0)		7	27	27	139
Addington et al. (2017);^66^ USA	352	N/K	2	Poor	18.52 (4.24)		318 (42.7)		4	14	81	263
**Register-based**												
Cederlöf et al. (2015);^47^ Sweden	216 700	1969-2009	2.4	Good	N/K	N/K	56.5%		476	196 524	557	19 143
Cheng et al. (2019);^67^ Taiwan	10 045	1997-2013	6.7	Good	<20: 376 (18.72%); 20-39: 833 (41.46%); ≥40: 800 (39.82%)	<20: 1504 (18.72). 20–39: 3332 (41.46); ≥40: 3200 (39.82)	1027 (51.12%)	4108 (51.12)	16	8020	115	1894
Kim et al. (2023);^68^ South Korea	10 036	2002-2013	5.35	Good	0-9: 1344 (13.4%); 10-19: 1972 (19.6%); 20-29: 1868 (18.6%); 30-39: 1700 (16.9%); 40-49: 1380 (13.8%); 50-59: 876 (8.7%); ⩾60: 896 (8.9%)		4848 (48.3)		17	7510	61	2448

Abbreviations: N/K, not known; OCSs, obsessive-compulsive symptoms; P, psychosis; +, presence of symptoms; −, absence of symptoms.

a Data are given by OCD group when available from manuscripts but otherwise given for the whole sample.

b The first three prospective studies give the average follow-up period and the remaining report the follow-up as a set period (eg, 2 years).

#### The RR of OCSs for Developing Psychosis in CHR Cohorts

**Figure 2:**
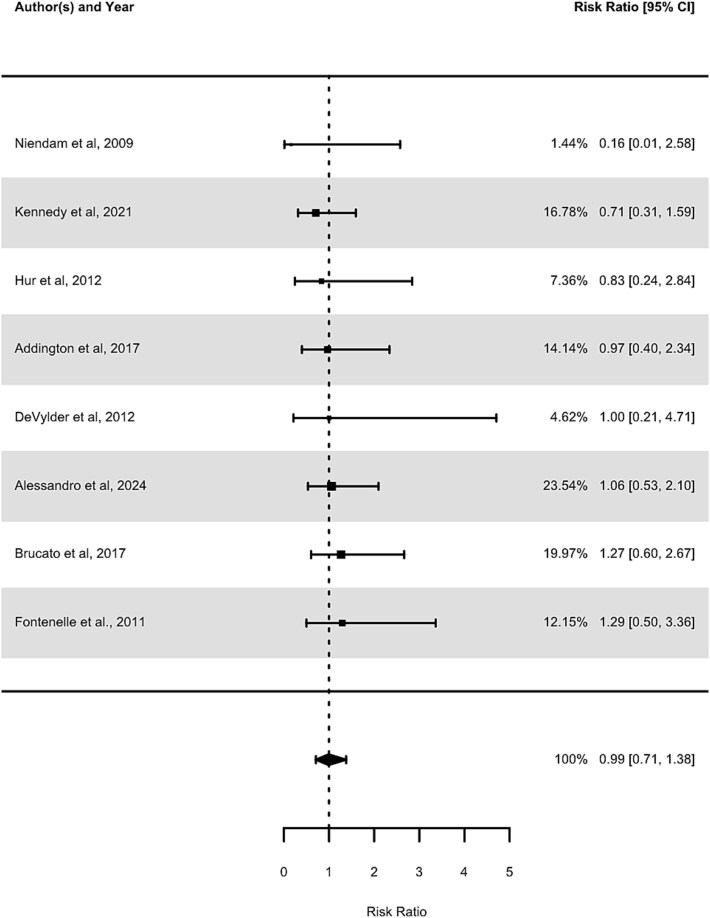
Forest Plot of Risk Ratio in CHR Studies

The relative RR of OCSs for developing psychosis in individuals at CHR from the 8 included studies was 0.99 (95% CI, 0.71-1.38, *P*=.95). The between-study heterogeneity was low, as measured by Cochran’s *Q* (*Q* = 3.14, df = 7, *P* = .87), *I*^2^ (*I*^2^ < 0.000%, 95% CI, 0-67.03), tau (*τ* < 0.000, 95% CI, 0-0.70), and tau-squared (*τ*^2^ < 0.000, 95% CI, 0-0.49). The prediction intervals (0.71-1.38) indicated that the RR in 95% of future studies would fall in a range around 1, indicative of no increase, or a small increase or decrease in the risk of developing psychosis for individuals with OCSs. No outliers were identified, and leave-one-out analysis revealed that none of the studies significantly influenced the findings. This RR suggests that people at CHR with OCSs do not have a significantly different risk of developing psychosis compared to those at CHR without OCSs, as shown in Figure 2.

#### The RR of OCSs for Developing Psychosis in Register-Based Cohorts

**Figure 3:**
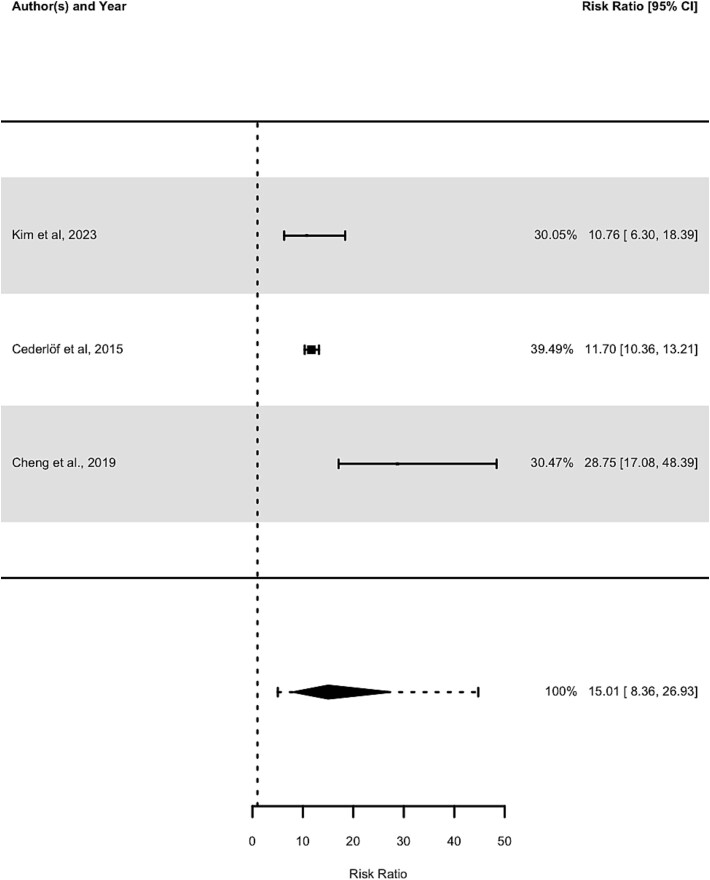
Forest Plot of Risk Ratio in Register-Based Studies

A meta-analysis of the 3 studies investigating the risk of developing psychosis in register-based cohorts found a relative RR of 15.01 (95% CI, 8.36-26.93, *P* < .001). However, between-study heterogeneity was high, as measured by Cochran’s *Q* (*Q* = 11.07, df = 2, *P* = .004), *I*^2^ (*I*^2^ = 85.1%, 95% CI, 37.14-99.67), tau (*τ* = 0.47, 95% CI, 0.15-3.41), and tau-squared (*τ*^2^ = 0.22, 95% CI, 0.02-11.65). The prediction intervals were wide (5.03-44.75), indicating there is uncertainty about the magnitude of the RR in future studies. No outliers were identified. This suggests that OCSs are associated with an approximately 15-fold increased risk of developing psychosis in register-based cohorts, as illustrated in Figure 3, although the small number of studies and high heterogeneity limit any conclusions.

### Quality Assessment

Three studies, all register-based, scored as good quality on the NOS and the remaining 8, all CHR studies, scored as poor (Supplementary Table S1). The interrater reliability found one discrepancy among the 24 rated items of the 3 studies, which was resolved through discussion and consensus. The data from these studies were drawn from national registries, which were representative of the general population, and all of them controlled for confounders with a matched control design. The retrospective design of all the studies meant that the follow-up duration was adequate.

The low overall score for the CHR studies was largely because of not scoring on the comparator section due to the absence of a control for confounders in the design or analysis. All the CHR studies scored highly in the selection section and were representative of CHR populations, having been recruited through typical routes with common inclusion and exclusion criteria. However, there was greater variation in the outcome section. All except one study had an adequate follow-up period of more than a year but only half reported drop-out rates. Two of the CHR studies independently assessed for exposure and two of the others had adequately followed up the cohort.

## Discussion

To the authors’ knowledge, this is the first systematic review and meta-analysis to investigate the risk of OCSs for developing psychosis in both CHR and population-level samples. The findings indicate that OCSs do not significantly increase the risk of psychosis in CHR cohorts but are associated with a 15-fold increased risk in population-level data. The results should be interpreted cautiously given the small number of studies (eight CHR and three population-level) and high heterogeneity observed in the population-level meta-analysis.

### Absence of Association between OCSs and Psychosis Risk in CHR Samples

The findings in the CHR analysis corroborate previous reviews showing that OCSs do not elevate the risk of developing psychosis,^45^^,^^46^ similar to findings of no association between transition and comorbid anxiety or depression in CHR youth.^14^ Although this meta-analysis had adequate power, based on power analysis and previous studies,^62^ comparable null results have been found for cannabis use^69^ and trauma^70–72^ where, although highly prevalent, there is no greater risk of transition. This suggests there may be a restriction of range in CHR samples that limits observable relationships between risk factors and transition.^73^

Measurement overlap may exist between OCSs and APS; for example, individuals with OCD often score similarly to those with psychosis on measures of hallucinations and delusions.^74^ The overlap may mean aspects of OCSs relevant to psychosis risk are already captured within APS ratings or that measurement error attenuates observable associations, resulting in OCSs adding little predictive value. Consequently, OCSs may not confer additional risk among individuals classified at CHR, despite shared vulnerabilities that contribute to a greater co-occurrence. While OCSs may not increase the transition risk, there is evidence that they are associated with greater overall psychopathology, more severe negative symptoms, and poorer functioning, highlighting their importance for treatment.^75^

### Elevated Risk of Psychosis in People with OCSs in Register-Based Cohorts

In contrast, national registers revealed that OCSs confer a substantial risk for developing psychosis, exceeding established risk factors such as mild traumatic brain injury (RR = 1.57, 95% CI, 1.28-1.91),^76^ urbanicity (OR = 2.37, 95% CI, 2.01-2.81),^77^ cannabis use (OR = 5.4, 95% CI, 2.81-11.31), for every day compared to not at all),^78^ childhood trauma (OR = 6.46, 95% CI, 4.37-9.53, for more than 5 exposure vs none),^79^ and socioeconomic status (OR = 8.10, 95% CI, 3.24-20.3, for 6 vs none).^80^ The relative risk is comparable to strong established risk factors such as familial risk (OR = 11.11, 95% CI, 1.45-85.02)^81^ and 22q11.2 deletion syndrome.^82^

As noted, there are shared genetic, neurobiological, and psychosocial mechanisms underlying OCSs and psychosis. It may be that OCSs represent a proximal risk factor, closer in the causal chain to frank psychosis, than distal factors such as trauma and familial history of the disorder, hence the larger RR.^83^ Common mechanisms may mediate this link, such as appraisal of intrusions as personally significant, leading to OCSs,^84^ whereas attributing them as external may contribute to hallucinations in psychosis.^85^ Similarly, fluctuations in insight,^86^ interpretation of thoughts as uncontrollable or dangerous,^87^ and the degree of superstitious beliefs may be factors that shift OCSs toward psychotic symptoms.^88^ However, future research should empirically investigate whether OCSs can transform into psychotic symptoms through these mechanisms.

### Differences between CHR and Register-Based Cohorts

One explanation for the difference between CHR and register-based cohorts is sampling biases arising from risk enrichment. Meta-analytic evidence indicates that CHR samples are substantially enriched for transition risk, increasing observations of development of psychosis^89^ but restricting informative variance in baseline risk and dominant predictors. Similarly, Berkson’s bias^90^ may obscure associations between OCSs and psychosis as a result of other strong predictors (eg, APS, negative symptoms^91^) being common pathways to study inclusion. Together, these factors may limit the ability to detect the effects of risk factors such as OCSs where pretest risk is high and variance is restricted.

Although the register-based studies typically had longer follow-up periods and larger sample sizes, follow-up alone is unlikely to account for the discrepancy. Most transitions in CHR cohorts occur within 3 years,^10^ with a median of 11 months,^92^ suggesting that the average follow-up period of the included CHR studies (2.6 years) would have captured the majority of transitions. In contrast, register-based studies may capture a broader range of individuals, including those with severe or untreated OCSs, which are associated with poorer outcomes,^93^ who present to community mental health teams or inpatient services. Additionally, detection may be better for individuals with OCSs and poor insight, who have a reduced likelihood of accessing services but a risk of developing delusions.^94^^,^^95^ Consequently, register-based designs may be more sensitive to earlier or alternative transition pathways that lose discriminative value at later enriched CHR stages. Conversely, the register-based studies were all retrospective, which may increase the risk of misclassification bias (eg, less standardized diagnostic protocols) and detection bias (eg, more intensive screening for psychosis in those with OCD than in those without).

Alternatively, service design and logistics may result in partially overlapping samples. For example, those at CHR with OCSs may be referred to specialist OCD services in the absence of CHR services or in the case of OCSs being a primary complaint. This would result in register-based cohorts including individuals who would be classified at CHR, and CHR cohorts underrepresenting individuals at CHR with OCSs. While CHR and register-based cohorts do not reflect entirely distinct clinical populations, they may reflect risk based on populations divided by real-world service structures.

### Limitations

Interpretations of the findings were constrained by methodological issues such as the small number of studies, particularly for register-based cohorts, which limited the power to assess the risk of publication bias or conduct metaregression. While the direction of effect was consistent in the register-based cohorts, the magnitude remains uncertain due to significant heterogeneity with very wide prediction intervals. Two studies with potential data (both at the population level) were excluded after failure to obtain data from the authors, although they appear to support the findings due to the large risk estimates. The quality ratings for all the CHR studies were poor, mostly due to the limited control for confounders in the design or analysis. Failure to adjust for demographic characteristics may distort associations between OCSs and psychosis, given the demographic influences on psychosis risk such as age, gender, and ethnicity.^96^

Additionally, conceptual issues such as defining OCSs, psychosis, and transition varied across studies, contributing to heterogeneity. For example, OCSs being measured categorically or dimensionally was inconsistent between studies. Subgroup analyses of OCS classification (ie, categorical or dimensional), recruitment strategy, and follow-up periods would have provided insight into the effects of the varied definitions. Unfortunately, the data available were insufficient to reliably account for this in the current analysis, but future research should aim to standardize definitions and provide adequate recruitment and follow-up details. The conceptual overlap between psychosis and OCSs also presents a clinical challenge where diagnoses in register-based cohorts may represent delayed recognition of psychotic symptoms initially thought to be OCSs, although the reverse may also be true.

### Clinical and Research Implications

The findings can be interpreted within a clinical staging model suggesting that OCSs confer a risk of psychosis in earlier stages but lose predictive value in later stages when multiple vulnerabilities are present.^97^ Traditional staging models assume a homotypic progression from subthreshold to full psychotic symptoms but there is evidence of psychosis occurring after nonpsychotic disorders and that around one-third of first episode of psychosis cases lack preceding subthreshold psychotic symptoms.^98^ Additionally, a higher population attributable fraction (that accounts for risk and incident rate) is observed in mood disorders compared to CHR groups.^99^ Together, this suggests that a broader identification strategy that captures both homotypic and heterotypic pathways may better reduce the overall burden of psychosis.^100^ For example, using the CHARMS criteria in EIP and secondary care services may improve detection efforts.

A study validating the CHARMS criteria did not include OCD in the criteria or outcomes but discussed the importance of including OCD in the criteria in future research due to the high prevalence in childhood.^101^ However, it is unclear whether specific OCD characteristics have differing effects on psychosis liability, such as if the transition risk varies between pediatric and adult-onset OCD cases, with earlier-onset cases thought to encompass a developmental subtype.^102^ There were insufficient data in the present review to determine mediators of transition risk, but research indicates earlier age of onset, OCD severity, compulsions, and functioning are associated with the greatest risk of psychosis.^40^ Further research into developmental perspectives of OCD and psychosis will help to improve the understanding of the clinical characteristics that affect those most at risk, and how to detect and intervene early.

In addition, there is evidence that psychotic symptoms may be underdetected in broader mental health services, with one study finding that two-thirds of a patient sample with OCD in a specialized anxiety disorder clinic met the criteria for a schizophrenia spectrum disorder.^103^ This might reflect individuals with psychosis coming into contact with services through affective complaints, termed a “lanthanic presentation,” where psychotic symptoms are obscured by other symptoms.^104^ This has led to concerns related to the validity of structured interviews in detecting psychosis.^105^ Also, there is evidence that psychotic symptoms are recorded in clinical notes but not recognized or diagnosed as psychotic disorders.^106^ Moreover, there is evidence that challenges in detection can result in a prolonged duration of untreated psychosis, particularly in community mental health teams, which may benefit from additional screening efforts that account for high-risk groups such as those with OCD.^107^

Early identification of OCSs and other distress could offer an opportunity for intervention before the emergence of psychosis. Evidence from OCD research shows that longer durations of untreated OCD predict poorer outcomes,^93^ paralleling findings in psychosis. Similarly, evidence suggests that the severity of OCD has a reverse-U shaped association with functioning in psychosis, with mild OCSs being protective but severe OCSs being related to poorer functioning.^108^ Furthermore, the severity and duration of untreated OCD may influence the transition risk. Additionally, it is yet to be empirically validated whether OCSs predict progression to CHR, as they are highly prevalent in CHR cohorts and linked to poorer outcomes.^109^^,^^110^ Future research should examine the phenomenology of overlapping symptoms (eg, intrusive guilt thoughts correlating with unusual thought content^111^) to determine under what conditions OCSs may mediate progression to psychosis. Similar research has begun to capture the developmental pathways of OCD from normative behavior to OC phenomena.^112^ An extension of this approach may help model the range of affective, neurobiological, and genetic factors explaining transition to psychosis.

Predictive models combining multiple variables, including known risk factors in CHR cohorts, such as cognitive and functioning deficits,^91^ alongside other demographic and clinical characteristics, show good prognostic accuracy and a promising area to refine risk prediction.^113^ However, while preventing transition to psychosis remains a valuable goal, it may be a challenging task, as focusing exclusively on this may neglect broader distress and impairment in individuals at CHR.^114^ This is important as most CHR youth seek treatment for anxiety and depression, rather than psychosis, and affective presentations predict poor functional outcomes, suggesting the importance of factors beyond transition.^115^ Future research should collect outcome data on transition, distress, and functioning to determine their associations with different risk profiles to improve treatment precision.

Future prospective studies should strengthen design quality by controlling for demographic factors, reporting detailed recruitment and follow-up data, and ensuring independent assessments. Clear, consistent definitions of variables, such as OCSs and psychosis, as well as clear reporting of categorical and dimensional measurement approaches, would also improve comparability across studies.

## Conclusion

In CHR samples, the risk of developing psychosis was comparable between individuals with and without OCSs, supporting previous findings. However, register-based studies indicate that people with OCD have a 15 times greater risk of developing psychosis. This exceeds established risk factors such as childhood trauma, cannabis use, and familial risk. Furthermore, OCSs may represent a proximal risk factor with direct influence on the development of psychotic symptoms, for example through cognitive or insight-related mechanisms. However, the conclusions are limited by the small number of studies and high heterogeneity among the register-based data. Differences between CHR and register-based cohorts might reflect methodological and sampling biases that restrict the representativeness of CHR studies. Conceptualizing the findings within a clinical staging model that accounts for multiple, heterotypic entry and exit pathways and integrating demographic, clinical, and cognitive variables may improve early identification of individuals at risk of developing psychosis.

## Supplementary Material

OCS_Psychosis_Supplement_sgag011
