# Supplementary material for: Do Obsessive-Compulsive Symptoms Increase the Risk of Developing Psychosis? A Systematic Review and Meta-analysis
Source: Schizophr Bull Open. 2026 May 12;7(1):sgag011. doi: 10.1093/schizbullopen/sgag011 (PMC13191126; doi:10.1093/schizbullopen/sgag011)
Supplement: OCS_Psychosis_Supplement_sgag011 [file ocs_psychosis_supplement_sgag011.docx]

Supplementary Table S1

Newcastle-Ottawa Scale for Cohort Studies Scores by Study

| Study | Overall | Selection | | | |  | Comparability |  | Outcome | | |
| --- | --- | --- | --- | --- | --- | --- | --- | --- | --- | --- | --- |
|  |  | 1 | 2 | 3 | 4 |  | 1 |  | 1 | 2 | 3 |
| **Prospective** |  |  |  |  |  |  |  |  |  |  |  |
| Fontenelle et al. (2011)^a^ | Poor | 1 | 1 | 1 | 1 |  | 0 |  | 0 | 1 | 1 |
| Niendam et al. (2009) | Poor | 1 | 1 | 1 | 1 |  | 0 |  | 0 | 0 | 1 |
| DeVylder et al. (2012)^a^ | Poor | 1 | 1 | 1 | 1 |  | 0 |  | 1 | 1 | 0 |
| Hur et al. (2012) | Poor | 1 | 1 | 1 | 1 |  | 0 |  | 0 | 1 | 0 |
| Alessandro et al. (2024) | Poor | 1 | 1 | 1 | 1 |  | 0 |  | 0 | 1 | 1 |
| Kennedy et al. (2021) | Poor | 1 | 1 | 1 | 1 |  | 0 |  | 0 | 1 | 0 |
| Brucato et al. (2017) | Poor | 1 | 1 | 1 | 1 |  | 2 |  | 0 | 1 | 0 |
| Addington et al. (2017) | Poor | 1 | 1 | 1 | 1 |  | 0 |  | 1 | 1 | 0 |
| **Retrospective** |  |  |  |  |  |  |  |  |  |  |  |
| Cederlöf et al. (2015)^a^ | Good | 1 | 1 | 1 | 1 |  | 2 |  | 1 | 1 | 1 |
| Cheng et al. (2019) | Good | 1 | 1 | 1 | 1 |  | 2 |  | 1 | 1 | 1 |
| Kim et al. (2023) | Good | 1 | 1 | 1 | 1 |  | 2 |  | 1 | 1 | 1 |

^a^ Studies screened by a second reviewer for inter-rater reliability
